# Multi-Objective Topology Optimization of a Compliant Parallel Planar Mechanism under Combined Load Cases and Constraints

**DOI:** 10.3390/mi8090279

**Published:** 2017-09-14

**Authors:** Gao Wang, Dachang Zhu, Ning Liu, Wei Zhao

**Affiliations:** 1 School of Information Science and Technology, Jinan University, Guangzhou 510632, China; twangg@jnu.edu.cn; 2 School of Mechanical and Electrical Engineering, Guangzhou University, Guangzhou 510006, China; 3 Key Laboratory of Disaster Forecast and Control in Engineering, Ministry of Education of China, Jinan University, Guangzhou 510632, China; zhaoweiyc@gmail.com

**Keywords:** multi-objective topological optimization, differential vector isomorphic mapping, 3-PRR planar micro displacement, SIMP

## Abstract

This paper focuses on a new type of configuration design of a compliant parallel mechanism (CPM) planar continuum structure and its characteristic analysis of vibration-inherent frequency for planar motion, which can suppress the impact of random vibration in ultra-precision positioning and manufacturing equipment and improve the inherent frequency response of the mechanism. Firstly, a vector-mapping isomorphism between the fully CPM and conventional isomorphic parallel mechanism was constructed with a kinematic differential Jacobian matrix. Then, the mathematical model of topology optimization was put forward considering the compromise programming on the static stiffness and mean vibration-inherent frequency of the mechanism as the design variable and the minimization of compliance as the objective function. A constraint of volume fraction was considered and multi-objective micro displacement mechanism topology optimization based on a prismatic-revolute-revolute (3-PRR) planar nano-positioning continuum structure was performed using the solid isotropic material with penalization (SIMP) technique, which combines the criteria of the optimization algorithm and the vector isomorphic mapping method. Multi-objective topology optimization of the continuum structure micro displacement mechanism was investigated and presented by optimizations with different initial rejection rates. The simulation results show that the stiffness and vibration suppression performance of the continuum structure were improved, whereas the positioning of differential kinematics characteristics of the 3-PRR micro displacement planar fully CPM and isomorphic prototype mechanism retain the same. The modal analysis also provides a rational configuration for the micro displacement mechanism dimensional design and its optimal modal parameters. The crossover oscillation in frequency response of the continuum structure was reduced and quickly converged in the optimization iterations. The performance of the optimized mechanism was verified by the experiments on a planar fully compliant micro displacement continuum structure based on Lead Zirconate Titanate (PZT) actuator.

## 1. Introduction

Nowadays, positioning mechanical components becomes very important for micro/nano applications such as cell manipulation, surgery, aerospace, micro fluidics, optical systems, micro machining and micro assembly etc. [1]. As an essential part of micro/nano manufacturing technology, high-precision positioning devices with controlled motions at sub-micron level is needed. The desired motion is provided with the deflection of these flexible joints called in the literature as “flexures” and the mechanisms, which are composed of flexures, instead of rigid joints are called “compliant mechanisms” [2]. The compliant parallel mechanisms/modules (CPMs) transmit motions/force by deflections of their compliant members and have characteristics of conventional CPMs. On the one hand, the CPMs have many advantages to be applied in high-precision applications providing high resolution, frictionless, smooth and continuous motion. On the other hand, the CPMs allow small displacements up to 0.01 μm with submicron accuracy, whose motion is insensitive to temperature changes due to their symmetrical structure, and provide weight reduction, compact structure and cheaper manufacturing cost than the high-precision mechanisms that use conventional rigid joints.

Parallel kinematic structures are mostly employed in micro positioning stages because of their advantages such as zero backlash, no need for lubrication, reduced wear, high precision, compact structure and monolithic configuration, but puzzled by their limited workspace, dexterity, non-linear kinematics, and difficult calculation of forward kinematics [3]. Fortunately, these drawbacks can be avoided for flexure-based compliant mechanisms because of their micro range motion. In addition, the kinematics can be assumed linear in the workspace range due to the small flexure displacements. The repeatability of these structures is eliminated with fewer flexures as there are no backlash or friction problems in the joints compared with rigid mechanisms. Various types of CPMs have been used in compliant positioning stages in the literature. These mechanisms are based on rigid body parallel prototypes. Many planar parallel compliant mechanisms have been designed based on triangular stages, in which, for compliant mechanisms, the most common kinematic structure 3-RRR is used [4]. Three linkages connected to each other with three revolute joints actuate the triangular stage. The end-effector has translation motion along *x*-*y* direction and a rotation about the *z* direction. This type of parallel kinematic structure amplifies the motion of the actuators. The revolute joints were replaced with flexure hinges, which were designed according to the desired parallel kinematic performance. Another triangular stage has 3-PRR kinematic structure, which is composed of 1 prismatic and 2 revolute joints and is used in [5] as a CPM.

There are three main approaches to designing CPMs: (a) Pseudo-Rigid-Body-Model synthesis methods proposed by Howell and Midha [6,7]; (b) Continuum Structure Optimization methods [8,9]; and (c) innovative design methods such as the constraint-based design approach, the building block approach [10,11], the screw theory-based approach and the freedom and constraint topology approach [12]. In addition, two main methods of solving force-displacement equations are concluded: (a) differential equation-based methods; and (b) energy methods, such as Castigliano’s theorem and virtual work principle. For the compliant mechanisms, there is no apparent boundary to identify the degrees-of-freedom (DOF) or the degrees-of-constraint (DOC) due to complex issues such as dimension, loads and motion range. The DOF of compliant mechanisms, especially in planar compliant mechanisms, were determined using the Pseudo-Rigid-Body-Model concept. [13] proposed an eigenscrew-based method to determine the DOF or compliance of compliant mechanisms. In addition, the constraint-based design approach, the screw theory-based approach and the freedom and constraint topology approach (FACT) [14,15] have been proposed to analyze the DOF and/or DOC of compliant mechanisms. Although the CPMs have great advantages to be used as micro positioning stages for high-precision applications, they are very sensitive to manufacturing tolerances and assembly errors. In analyzing the DOF of a compliant mechanism quantitatively, it is difficult to realize the spatial multi-DOF micro/nano positioning operation with the compliant mechanism. Concerned with this issue, some researchers designed a new type of fully compliant parallel mechanism, which replaces the flexible hinge with a rigid hinge, to integrate conventional parallel spatial multi-DOF mechanism characteristics into compliant mechanism [16,17]. However, the design often depends on the designer’s experience, which will lead to the diversity of flexible hinge configuration, namely, only the hinges are flexible but the other parts are still rigid. The kinematics and dynamics analysis of rigidity-flexibility hybrid pattern have become more complicated and the overall rigidity of compliant mechanism is lacking. Therefore, it is hard to effectively suppress the disturbance of environmental random vibration and guarantee precision positioning and machining accuracy. Moreover, it is also difficult to satisfy the requirements of overall rigidity and precision requirement [4].

Hao and Kong [5,18,19] performed a nonlinear analysis of spatial CPM-based multi-beam modules and proposed a normalization-based approach for the mobility analysis of this class of spatial compliant parallel modules (CPMs) to address the dimensional-inhomogeneity issue of motion/load. The normalization strategy (non-dimensional/homogeneous measures) can unify the dimensions of compliant mechanisms and be used in modeling and the design of compliant/flexural mechanisms. Zhang et al. [20] researched the coupling characteristics of elastic motions and rigid body motions for a 3-PRR planar parallel manipulator with three flexible intermediate links to eliminate unwanted flexible vibrations, and the designed lightweight manipulator had the capability of moving swiftly but was deprived of practicability.

Among the analytical methods used for structure innovation design, the evolutionary structural optimization (ESO) method is a good choice for its simplicity and ease of implementation with a general finite element software. Xie and Steven roposed the concept of gradually removing redundant material in the method of finite element analysis to achieve an optimal design [21]. It also has been demonstrated for more practical problems with multiple load cases or to optimize for structural frequency. A procedure to deal with the problems of stiffness constraint and a modified rejection ratio for multiple load case ESO has been developed for better results. It was also found that the element size had a significant effect on the history of minimum stresses and volume reduction as well. The optimized structure topology is mainly controlled by element size. The choice of the initial rejection ratio and the evolutionary rate also have significant influence on structure topology. Some improved ESO methods based on SIMP are proposed to reduce the step length of evolution for improving the accuracy of sensitivity analysis. The SIMP method is employed to describe the relationship between the relative density and stiffness of elements, the relative densities of elements are taken as the design variables, and the mean compliance is selected as the objective function to prevent checkerboards and eliminate mesh independency [22,23]. The proposed strategy firstly obtained a rough design of the structural topology by eliminating less-stressed material, then computed the stiffness and frequency, and analyzed local stress at the last step.

It is revealed that using the criteria of optimization algorithm and vector isomorphic mapping methods will cause diversity result changes under a variety of topology and constraint conditions. In the optimization iterations, elements whose relative densities were less than or equal to rejection ratio were removed from the design domain and all remaining elements were entered into the next iteration. Based on the work in [24], Wang and Zhu proposed a 3-RRR type CPM for displacement and the work in [25] also presented a new sensitivity reanalysis of static displacement for arbitrary changes of design variables, which is a key step for this research. This type of CPM has good characteristics such as kinematostatic decoupling and enlarging the range of motion. The homologous 3-PRR mechanism in this type of continuum structure optimized under topology constraints will obtain more benefit for practice.

Based on the above methods, this paper further investigates some sorts of continuum structures under special load cases, which cannot be optimized smoothly, or where the later optimization of this continuum structure is terminated abruptly when the removal of material increases excessively. In the optimization iterations, elements whose relative densities were less than or equal to the rejection ratio were removed from the design domain and all remaining elements were retained into the next iteration. It can timely and flexibly adjust the criterion of inefficient material removal. This procedure is based on systematic and gradual removal of the elements with lower stress compared with the maximum stress of the structure. Nevertheless, the method neither directly reflects the kinematics and dynamics performance nor effectively meets high-precision positioning requirements. Fortunately, it can be processed with all the flexible hinges being integrated into a continuum structure using WEDM (Wire-cut Electrical Discharge Machining). The integrated CPMs thus are obtained [26]. There remains a problem of how to configure flexible hinges in the integrated CPMs. It is necessary to put forward a new design method for CPMs in a continuum structure to obtain high stiffness, mechanism uniqueness and multi-DOF kinematics performance. A practical approach for the new CPMs synthesis uses topology optimization theory with modal parameters, including boundary constraints and load case conditions and maximizing integral stiffness. Several studies have been conducted on the flexibility minimizing with a single objective orient optimization [27,28,29], which adopted a compliant mechanism synthesis method. However, the coupling between the flexibility and vibration-inherent frequency is ignored, and the designed mechanism structure cannot actively suppress the influence of mechanical operation on accuracy due to the random vibration under load case conditions. According to the kinematic characteristics of high-precision micro/nano positioning displacement worktable, a novel planar mechanical form synthesis method for a fully CPM is proposed in this paper. Based on the output criteria of the spatial vector constraint in the parallel mechanism prototype, a multi-objective optimization function was put forward to maximize the limb stiffness and the vibration-inherent frequency. As a peristaltic movement illustration, a 3-PRR planar fully compliant precision positioning micro displacement worktable is considered. The Jacobian matrix of the vector motion is established as multi-objective topology optimization under output-equivalent criteria, and a fully CPM topology optimization model was constructed using the continuum SIMP method [24,30] and the ordered multi-material SIMP interpolation could solve multi-material topology optimization problems [31]. An analysis of the characteristic of CPMs’ kinematics indicates that the main issue is rationally allocating the weights of the compromise programming method and adopting a mean inherent frequency method. The static stiffness, stress distribution, the first 6 modes of vibration-inherent frequency and its modal parameters can be obtained. The experimental results demonstrate that the proposed method is able to obtain a perfect topology mechanism and satisfy positioning displacement accuracy requirement.

This paper is organized as below. The fundamental theory regarding the Jacobian equation construction by vector isomorphism mapping is described in Section 2. The multi-objective topology optimization model of 3-PRR planar fully CPM is depicted in Section 3. The processing of multi-objective optimization and analysis with Hyperworks^®^ (Altair Engineering, Inc., Troy, MI, USA) and Optistruct^®^ (Altair Engineering, Inc.) respectively is illustrated and discussed in Section 4. Some experiment results are listed in Section 5. The conclusion is given in Section 6.

## 2. Jacobian Equation Construction by Vector Isomorphism Mapping

### 2.1. Conventional 3-PRR Planar Parallel Manipulator

**Figure 1:**
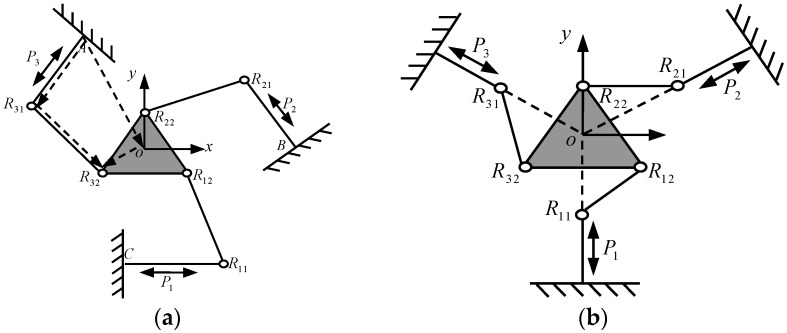
Structure of 3-PRR planar parallel prototype manipulator, (**a**) active pairs intersected with each other; (**b**) active pairs intersected at a point.

The conventional 3-PRR planar CPM includes a moving platform, fixed platform and 3 limbs connecting with them. Each limb is constituted of 1 prismatic (P) pair and 2 revolute (R) pairs, as shown in Figure 1. According to the difference of active pair P planar form, the 3-PRR parallel mechanism can perform 2 movement forms that are two translations or two translations and one rotation in the plane, as shown in Figure 1a,b respectively. The form of Figure 1a is adopted in this paper.

### 2.2. Isomorphic Vector Jacobian Matrix

The planar fully CPMs should be consistent in terms of prototypical mechanism movement performance, namely isomorphic properties. The kinematic Jacobian matrix reveals the mapping relations between manipulating space and joints space. The isomorphic properties should have the same kinematic Jacobian matrix between prototype mechanism and topology optimization results. The kinematic vector Jacobian matrix of prototypical 3-PRR planar CPMs thus can be solved, and set as isomorphic vector equation constraints of mechanism topology optimization.

**Figure 2:**
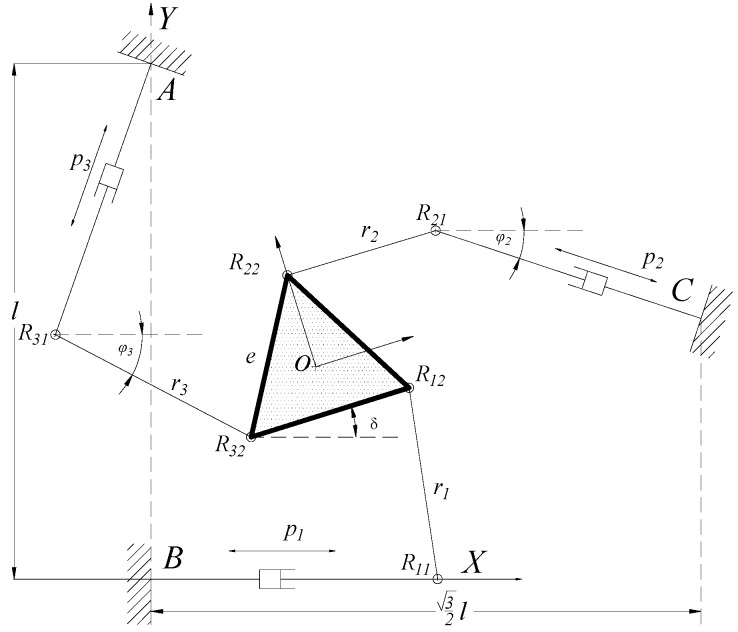
3-PRR planar parallel prototype mechanism.

The prototype mechanism is shown in Figure 2. Vector equation of the limbs *AR*_31_*R*_32_ can be described as

(1) $$\vec{AO}+\vec{OR_{32}}=\vec{AR_{31}}+\vec{R_{31}R_{32}}$$

Differentiating Equation (1) with respect to design variables, it can be obtained

(2) $$v_{o}+\dot{\phi }\cdot \left(k\times \vec{OR_{32}}\right)={\dot{p}}_{3}\cdot \left(k\times \vec{AR_{31}}\right)+\left({\dot{p}}_{3}+{\dot{\psi }}_{3}\right)\cdot \left(k\times \vec{R_{31}R_{32}}\right)$$

where $\dot{\phi }$ is the rotational angle around the center of the moving platform with *R*_32_; ${\dot{p}}_{3}$ is the linear driving velocity of prismatic pair; ${\dot{\psi }}_{3}$ is the rotational angle of limb *R*_31_*R*_32_, *k* is the unit vector in *x* direction; the cross product in Equation (2) represents projection direction towards *x* direction.

Considering the dot product between two sides of Equation (2) with *R*_31_*R*_32_, it can be obtained

(3) $$\vec{R_{31}R_{32}}\cdot v_{o}+\dot{\phi }\cdot k\cdot \left(\vec{OR_{32}}\times \vec{R_{31}R_{32}}\right)={\dot{p}}_{3}\cdot k\cdot \left(\vec{AR_{31}}\times \vec{R_{31}R_{32}}\right)$$

Similarly, we can obtain the vector equations of two limbs *R*_11_*R*_12_ and *R*_21_*R*_22_, and rewrite them with matrix form as

(4) $$J_{x}\cdot \dot{x}=J_{q}\cdot \dot{q}$$

where

$$\begin{array}{l} J_{x}=\left[\begin{array}{ccc} {\vec{R_{11}R_{12}}|}_{x} & {\vec{R_{11}R_{12}}|}_{y} & {\vec{OR_{12}}|}_{x}{\cdot \vec{R_{11}R_{12}}|}_{y}-{\vec{OR_{12}}|}_{y}{\cdot \vec{R_{11}R_{12}}|}_{x}\\ {\vec{R_{21}R_{22}}|}_{x} & {\vec{R_{21}R_{22}}|}_{y} & {\vec{OR_{22}}|}_{x}{\cdot \vec{R_{21}R_{22}}|}_{y}-{\vec{OR_{22}}|}_{y}{\cdot \vec{R_{21}R_{22}}|}_{x}\\ {\vec{R_{31}R_{32}}|}_{x} & {\vec{R_{31}R_{32}}|}_{y} & {\vec{OR_{32}}|}_{x}{\cdot \vec{R_{31}R_{32}}|}_{y}-{\vec{OR_{32}}|}_{y}{\cdot \vec{R_{31}R_{32}}|}_{x} \end{array}\right],\\ J_{q}=\left[\begin{array}{ccc} {\vec{CR_{11}}|}_{x}{\cdot \vec{R_{11}R_{12}}|}_{y}-{\vec{CR_{11}}|}_{y}{\cdot \vec{R_{11}R_{12}}|}_{x} & 0 & 0\\ 0 & {\vec{BR_{21}}|}_{x}{\cdot \vec{R_{21}R_{22}}|}_{y}-{\vec{BR_{21}}|}_{y}{\cdot \vec{R_{21}R_{22}}|}_{x} & 0\\ 0 & 0 & {\vec{CR_{31}}|}_{x}{\cdot \vec{R_{31}R_{32}}|}_{y}-{\vec{CR_{31}}|}_{y}{\cdot \vec{R_{31}R_{32}}|}_{x} \end{array}\right] \end{array}$$

Transforming Equation (4) with pre-multiplication of the inverse matrix of *J_x_* on both sides, we can obtain the kinematics Jacobian equation

(5) $$\dot{x}=J_{D}\dot{q}$$

where $J_{D}=J_{x}^{-1}\cdot J_{q}$; $\dot{x}={\left[\begin{array}{ccc} v_{ox} & v_{oy} & \dot{\delta } \end{array}\right]}^{T}$ denotes the displacement velocity in *x*-*y* directions and palstance revolved around *z* direction in the moving platform respectively; $\dot{q}={\left[\begin{array}{ccc} {\dot{p}}_{1} & {\dot{p}}_{2} & {\dot{p}}_{3} \end{array}\right]}^{T}$ denotes the displacement velocity of the three prismatic pairs; ${\cdot |}_{x}$ and ${\cdot |}_{y}$ denote vector components in *x*-*y* directions.

### 2.3. Isomorphic Vector Kinematic Differential Jacobian Equation

To obtain the same differential movement performance of topological optimization of fully CPMs as conventional isomorphic parallel mechanisms, the topological optimization constraints should be a conventional parallel mechanism differential movement Jacobian matrix. In this paper, the 3-PRR planar CPM is set as an example to illustrate and design isomorphic 3-PRR planar fully CPMs with multi-objective topological optimization. The 3-PRR CPMs is shown in Figure 2. According to the vector isomorphic mapping method, Jacobian matrix *J_D_* and differential kinematic of CPMs can be obtained, which is the differential solution with respect to variables in matrix.

When the parameter varies, namely $\varphi_{1}\to \varphi_{1}+\Delta \varphi_{1}$, the constraint equations of limb *CR*_11_*R*_12_ can be written as

(6) $$\left\{ \begin{array}{l} {R_{12}|}_{x}=p_{1}+r_{1}\cdot \cos\varphi_{1}\\ {R_{12}^{\prime }|}_{x}=p_{1}^{\prime }+r_{1}\cdot \cos(\varphi_{1}+\Delta \varphi_{1})\\ {R_{12}|}_{y}=r_{1}\cdot \sin\varphi_{1}\\ {R_{12}^{\prime }|}_{y}=r_{1}\cdot \sin(\varphi_{1}+\Delta \varphi_{1}) \end{array}\right.$$

where $\cos\Delta \varphi_{1}=1$, $\sin\Delta \varphi_{1}=\Delta \varphi_{1}$, $\Delta x={R_{12}|}_{x}-{R_{12}^{\prime }|}_{x}$, $\Delta y={R_{12}|}_{y}-{R_{12}^{\prime }|}_{y}$. With the micro/nano kinematic characteristic and principle of equivalent infinitesimal, we can obtain

(7) $$\left\{ \begin{array}{l} \Delta x=r_{1}\cdot \Delta \varphi_{1}\cdot \sin\varphi_{1}-\Delta p_{1}\\ \Delta y=-r_{1}\cdot \Delta \varphi_{1}\cdot \cos\varphi_{1} \end{array}\right.$$

The kinematics variables of limb *CR*_11_*R*_12_ can be described as

(8) $$\Delta x\cdot \cos\varphi_{1}+\Delta y\cdot \sin\varphi_{1}+\Delta p_{1}\cdot cos\varphi_{1}=0$$

Similarly, with $\varphi_{2}\to \varphi_{2}+\Delta \varphi_{2}$, the constraint equations of limb *BR*_21_*R*_22_ can be described as

(9) $$\left\{ \begin{array}{l} {R_{22}|}_{x}={R_{12}|}_{x}-e\cdot \cos(\frac{\pi }{3}-\varphi )=\frac{\sqrt{3}}{2}\cdot l-\frac{1}{2}\cdot p_{2}+r_{2}\cdot \cos\varphi_{2}\\ \;{R_{22}|}_{y}={R_{12}|}_{y}+e\cdot \sin(\frac{\pi }{3}-\varphi )=\frac{1}{2}\cdot l+\frac{\sqrt{3}}{2}\cdot p_{2}+r_{2}\cdot \sin\varphi_{2}\\ {R_{22}^{\prime }|}_{x}={R_{12}|}_{x}-\frac{1}{2}\cdot e\cdot \cos\left(\delta +\Delta \delta \right)-\frac{\sqrt{3}}{2}\cdot e\cdot \sin\left(\delta +\Delta \delta \right)=\frac{\sqrt{3}}{2}\cdot l-\frac{1}{2}\cdot r_{2}^{\prime }+p_{2}\cdot \cos\left(\varphi_{2}+\Delta \varphi_{2}\right)\\ {R_{22}^{\prime }|}_{y}={R_{12}|}_{y}+\frac{\sqrt{3}}{2}\cdot e\cdot \cos\left(\delta +\Delta \delta \right)-\frac{1}{2}\cdot e\cdot \sin\left(\delta +\Delta \delta \right)=\frac{1}{2}\cdot l+\frac{\sqrt{3}}{2}\cdot r_{2}^{\prime }+p_{2}\cdot \sin\left(\varphi_{2}+\Delta \varphi_{2}\right) \end{array}\right.$$

and

(10) $$\left\{ \begin{array}{l} \Delta x=\frac{1}{2}\cdot \Delta r_{2}+p_{2}\cdot \Delta \varphi_{2}\cdot \sin\varphi_{2}-\frac{1}{2}\cdot e\cdot \Delta \delta \cdot \sin\delta +\frac{\sqrt{3}}{2}\cdot e\cdot \Delta \delta \cdot \cos\delta \\ \Delta y=\frac{\sqrt{3}}{2}\cdot \Delta r_{2}-p_{2}\cdot \Delta \varphi_{2}\cdot \cos\varphi_{2}+\frac{\sqrt{3}}{2}\cdot e\cdot \Delta \delta \cdot \sin\delta +\frac{1}{2}\cdot e\cdot \Delta \delta \cdot \cos\delta \end{array}\right.$$

The kinematics variables of limb *BR*_21_*R*_22_ can be described as

(11) $$\Delta x\cdot \cos\varphi_{2}+\Delta y\cdot \sin\varphi_{2}-\frac{\sqrt{3}}{2}\cdot e\cdot \Delta \delta \cdot \cos(\delta -\varphi_{2})+\frac{1}{2}\cdot e\cdot \Delta \delta \cdot \sin(\delta -\varphi_{2})-\frac{1}{2}\cdot \Delta p_{2}\cdot \cos\varphi_{2}+\frac{\sqrt{3}}{2}\cdot \Delta p_{2}\cdot \sin\varphi_{2}=0$$

With $\varphi_{3}\to \varphi_{3}+\Delta \varphi_{3}$, the constraint equations of limb *AR*_31_*R*_32_ can be described as

(12) $$\left\{ \begin{array}{l} {R_{32}|}_{x}={R_{12}|}_{x}-e\cdot \cos\delta =-\frac{\sqrt{3}}{2}\cdot p_{3}+r_{3}\cdot \cos\varphi_{3}\\ {R_{32}|}_{y}={R_{12}|}_{y}-e\cdot \sin\delta =l-\frac{1}{2}\cdot p_{3}-r_{3}\cdot \cos(\varphi_{3}+\Delta \varphi_{3})\\ {R_{32}^{\prime }|}_{x}={R_{12}|}_{x}-e\cdot \cos(\delta +\Delta \;\delta )=-\frac{\sqrt{3}}{2}\cdot p_{3}^{\prime }+r_{3}\cdot \cos(\varphi_{3}+\Delta \varphi_{3})\\ {R_{32}^{\prime }|}_{y}={R_{12}|}_{y}-e\cdot \sin(\delta +\Delta \delta )\;=l-\frac{1}{2}\cdot p_{3}^{\prime }-r_{3}\cdot \sin(\varphi_{3}+\Delta \varphi_{3}) \end{array}\right.$$

So

(13) $$\left\{ \begin{array}{l} \Delta x=\frac{\sqrt{3}}{2}\cdot \Delta p_{3}+r_{3}\cdot \Delta \varphi_{3}\cdot \sin\varphi_{3}-e\cdot \Delta \delta \cdot \sin\delta \\ \Delta y=\frac{1}{2}\cdot \Delta p_{3}+r_{3}\cdot \Delta \varphi_{3}\cdot \cos\varphi_{3}+e\cdot \Delta \delta \cdot \cos\delta \end{array}\right.$$

The kinematics variables of limb *BR*_21_*R*_22_ can be described as

(14) $$\Delta x\cdot \cos\varphi_{3}-\Delta y\cdot \sin\varphi_{3}+r\cdot \Delta \delta \cdot \sin\delta \cdot \cos\varphi_{3}-r\cdot \Delta \delta \cdot \cos\delta \cdot \sin\varphi_{3}-\frac{\sqrt{3}}{2}\cdot \Delta p_{3}\cdot \cos\varphi_{3}+\frac{1}{2}\cdot \Delta p_{3}\cdot \sin\varphi_{3}=0$$

Assuming that the displacements of each limb are equal to infinity and defined as $\varphi_{i}\to \varphi_{i}+\Delta \varphi_{i}$, *i* = 1, 2, 3, then Equations (8), (11) and (14) can be rearranged and simplified as kinematics Jacobian matrix *Q* with infinite displacements. It is revealed that the vector continuous mapping relation [*p*_1_ *p*_2_ *p*_3_]*^T^* = [*Q*] [*x y δ*]*^T^* of 3-PRR parallel prototype mechanism between the limbs and task displacement by Equation (15)

(15) $$\left[\begin{array}{c} \Delta p_{1}\\ \Delta p_{2}\\ \Delta p_{3} \end{array}\right]=\left[\begin{array}{ccc} -1 & \tan\varphi_{1} & 0\\ \frac{\cos\varphi_{2}}{\cos\left(\varphi_{2}+\frac{\pi }{3}\right)} & \frac{\sin\varphi_{2}}{\cos\left(\varphi_{2}+\frac{\pi }{3}\right)} & \frac{e\cdot \sin\left(\delta -\varphi_{2}-\frac{\pi }{3}\right)}{\cos\left(\varphi_{2}+\frac{\pi }{3}\right)}\\ \frac{\cos\varphi_{3}}{\sin\left(\frac{\pi }{3}-\varphi_{3}\right)} & \frac{\sin\varphi_{3}}{\sin\left(\frac{\pi }{3}-\varphi_{3}\right)} & \frac{e\cdot \sin(\delta -\varphi_{3})}{\cos\left(\frac{\pi }{3}-\varphi_{3}\right)} \end{array}\right]\cdot \left[\begin{array}{l} \Delta x\\ \Delta y\\ \Delta \delta \end{array}\right]=\left[Q\right]\cdot \left[\begin{array}{l} \Delta x\\ \Delta y\\ \Delta \delta \end{array}\right]$$

where [*x y δ*]*^T^* is the prismatic pair representing kinematic input, [*p*_1_ *p*_2_ *p*_3_]*^T^* is the kinematic output, and *Q* is the Jacobian matrix. In the process of topology optimization, calculating *J* = [*Q*]^−1^ to reflect the differential movement isomorphic properties of 3-PRR planar fully CPMs.

## 3. 3-PRR Planar Fully CPMs Multi-Objective Topological Optimization Model

### 3.1. Static Stiffness Topological Optimization Model

The maximum structural stiffness topological optimization problem is how to obtain the best material distribution of a constructed mechanism in a given field. According to the input/output mapping relation and SIMP method of the parallel mechanism, we construct a 3-PRR topology optimization model with the material density as design variable, minimizing the flexibility as the objective function and the volume ratio as the constraint condition. In optimization iterations, the element-intermediate density varies between 0 and 1 with a penalty factor and an element-relative density, thus a {0, 1} discrete variable optimization model can be obtained [32].

The relationship between the initial and optimized modulus of elasticity can be written as

(16) $$E_{e}(\rho )=\rho_{e}^{p}\cdot (E_{0}-E_{\min})+E_{\min},0<\rho_{\min}\leq \rho_{e}\leq 1$$

where *E*_ε_ is *e*-*i*th element modulus of elasticity, *ρ*_e_ is *e*-*i*th element-relative density, *E*_0_ is the initial elastic modulus of material, *E*_min_ is the element modulus of elasticity of the hollow material taken as *E*_0_/1000 for the numerical stability, *ρ*_min_ is the minimum density with empty material, and *p* is penalty factor with constant value 3. In 2-dimension optimization, the penalty factor codomain of intermediate density material should satisfy

(17) $$p\geq \max\left\{\frac{2}{1-\mu_{0}},\frac{4}{1+\mu_{0}}\right\}$$

where *μ*_0_ is the Poisson’s ratio of given material with constant value 0.3 [33].

According to the Jacobian matrix of micro displacement in 3-PRR fully CPMs, continuous mapping topology optimization SIMP model can be written as

(18) $$\left\{ \begin{array}{l} \min\;\;\;\;\;\;\;\;\;\;C=\sum_{i=1}^{3}\sum_{j=1}^{3}{\tilde{U}}_{j}^{T}\cdot K\cdot U_{i}=\sum_{e=1}^{N}\sum_{i=1}^{3}\sum_{j=1}^{3}\;{\tilde{U}}_{ej}^{T}\cdot \rho_{e}{}^{p}\cdot K_{e}\cdot U_{ei}\\ s.t.\;\;\;\;\;\;\;\;\;\;\;\;\left\{K\cdot {\tilde{U}}_{j}=F_{j},\;\;\;K\cdot U_{i}=F_{i},\;\;\;\;F_{j}=Q_{ij}\cdot F_{i}\;\;\right\}\;\;\;\;\;\;\;i=1,2,3.\;\;\;\;\;j=1,2,3.\\ \forall :\to \;\;\;\int_{\Omega }\rho_{e}d\Omega \leq V,\;\;\;\;\;0<\rho_{\min}\leq \rho_{e}\leq 1\;\;\;\;\;\;\;\;\;\;\;\;e=1,2,\cdots ,N. \end{array}\right.$$

In Equation (18), ${\tilde{U}}_{j}$ is the companion displacement vector, *K* is the whole post optimality stiffness of creep structure, *U_i_* is the displacement under the actual load, *F_i_* is the *i*th actual load, *U_ei_* is the element-displacement vector under the *i*th actual load, *F_j_* is *j*th virtual load, ${\tilde{U}}_{ej}$ is the element-displacement vector under the *j*th virtual load, *K_e_* is the element stiffness, *V* is the permitted material volume in design codomain. To avoid the whole stiffness singular matrix, we assume $\rho_{\min}=0.001$, and *Q_ij_* is vector isomorphic mapping matrix with respect to kinematic output ${\dot{p}}_{i}$

(19) $$Q_{ij}=\frac{\partial f(v_{ox},v_{oy},\dot{\delta })}{\partial {\dot{p}}_{1}}d{\dot{p}}_{1}+\frac{\partial f(v_{ox},v_{oy},\dot{\delta })}{\partial {\dot{p}}_{2}}d{\dot{p}}_{2}+\frac{\partial f(v_{ox},v_{oy},\dot{\delta })}{\partial {\dot{p}}_{3}}d{\dot{p}}_{3}$$

The topological optimization objective function of a 3-PRR fully compliance parallel mechanism in single load case and multi-input compromise programming method is given by

(20) $$\underset{\rho }{\min}C(\rho )={\left\{\sum_{i=1}^{m}{\left[\omega_{i}\cdot \frac{C_{i}(\rho )-C_{i}^{\min}}{C_{i}^{\max}-C_{i}^{\min}}\right]}^{p}\right\}}^{\frac{1}{p}}$$

where *m* is the element of load case, *ω_i_* is the *i*th load case weighting, *p* is the penalty factor with constant value 3, *C_i_*(*ρ*) is the flexibility objective function of *i*th load case, $C_{i}^{\max}$ and $C_{i}^{\min}$ are the maximum and minimum of *i*th load case flexibility objective function respectively.

### 3.2. Dynamic Inherent Frequency Topological Optimization Model

Dynamic inherent frequency optimization sets maximizing some significant frequencies in low order as objective functions, and topological optimization design will be performed with structural volume fraction as the constraint condition. However, in the optimal iterations, the oscillation problem of objective function will occur in the stage of neighbor higher-order eigenvalue falling to a lower order due to individual structural element material removable performance. To overcome the oscillation phenomenon in some orders frequencies, the objective function of dynamic inherent frequency topology optimization is set to mean frequency eigenvalue *Λ*, which proposed by [34]

(21) $$\Lambda =\{\begin{array}{ll} \Lambda_{0}+{\left(\frac{1}{s}\cdot \sum_{i=1}^{m}\omega_{i}\cdot {\left(\lambda_{i}-\lambda_{0}\right)}^{n}\right)}^{\frac{1}{n}} & if(n\neq 0)\exists n=\pm 1,\pm 2,\cdots \\ \Lambda_{0}+\exp\left(\frac{1}{s}\cdot \sum_{i=1}^{m}\omega_{i}\cdot \ln|\lambda_{i}-\lambda_{0}|\right) & if(n=0). \end{array}$$

Here *Λ* is mean frequency eigenvalue, *λ_i_* is *i*th eigenvalue, *λ*_0_ and *s* are the given parameters, *ω_i_* is the weights of *i*th eigenvalue, and *m* is the order value of optimized low-order eigenvalue. The exponential *n* is the factor of frequency determination. When *n* is a negative odd, the inherent frequency of *Λ* specified orders should be maximized. When *n* is a negative even, the difference between specified order inherent frequency and given inherent frequency should be maximized.

In this paper, the objective function based on mean frequency eigenvalue maximization is conducted with the constraint conditions of volume fraction for the topology optimization of 3-PRR fully compliant parallel mechanism. To solve optimization conveniently, we set *n* constant value −1 referring to literature [22]. The optimization model is written as follows

(22) $$\left\{ \begin{array}{l} \underset{\rho_{1},\rho_{2},\cdots ,\rho_{n}}{\max}\left\{\Lambda |{}_{\rho_{i}}=\lambda_{0}+s\cdot {\left(\sum_{i=1}^{m}\frac{\omega_{i}}{\lambda_{i}-\lambda_{0}}\right)}^{-1}\right\}\;\;.\;\;\;\\ s.t.\;\;\;\;\;\;\;\;\;\;\;\;\;\{\begin{array}{ll} (K-\lambda_{i}M)\cdot \left\{\delta_{i}\right\}=0 & i=1,2,\cdots ,D_{g}\\ \sum_{e=1}^{n}V_{e}\cdot \rho_{e}-V_{*}\leq 0 & V_{*}=\alpha \cdot V_{0}\\ 0<\rho_{\min}\leq \rho_{e}\leq 1 & e=1,2,\cdots ,N \end{array}\;\;\;\;\;\;\; \end{array}\right.$$

where *K* is the whole stiffness matrix, *M* is the whole mass matrix, {*δ_i_*} is the eigenvector with *i*th eigenvalue, and *D_g_* is the whole DOFs of finite element model of optimization mechanism.

### 3.3. Weighted Stiffness and Frequency Eigenvalue Multi-Objective Topological Optimization Model

An optimization-weighted model linearly grouped with objective function based on compromise programming and mean frequency eigenvalue can be obtained, which is combined static flexibility minimization in Equation (20) with dynamic inherent frequency maximization in Equation (22). The multi-objective topological optimization function of 3-PRR fully compliant parallel mechanism can be described as Equation (23) by the integrated two single-objective functions

(23) $$\underset{\rho }{\min}F(\rho )={\left\{\omega^{2}\cdot {\left[\sum_{i=1}^{m}\omega_{i}\cdot \frac{C_{i}(\rho )-C_{i}^{\min}}{C_{i}^{\max}-C_{i}^{\min}}\right]}^{2}+{\left(1-\omega \right)}^{2}\cdot {\left[\frac{\Lambda_{\max}-\Lambda (\rho )}{\Lambda_{\max}-\Lambda_{\min}}\right]}^{2}\right\}}^{\frac{1}{2}}$$

where the *F*(*ρ*) is the integrated objective function, *ω* is the weighted objective function of flexibility and inherent frequency, *Λ*_max_ and *Λ*_min_ are the maximum and minimum of inherent frequency respectively, which are convenient to non-dimensionalize.

### 3.4. Multi-Objective Optimization Criteria and Iterative Formula

Differentiating (23) with respect to relative density *ρ_e_* in multi-objective functions with gradient-iterative method yields

(24) $$\frac{\partial F}{\partial \rho }d\rho =\frac{\partial C}{\partial \rho_{e}}d\rho_{e}+\frac{\partial \Lambda }{\partial \rho_{e}}d\rho_{e}$$

Assuming that the input and output are unconcerned with design variables, and differentiating the total stiffness matrix *K*, the total mass matrix *M*, and the *m*th eigenvalue *λ_m_* with respect to design variables *ρ_e_*, the sensitivity of constraint function can be derived as

(25) $$\left\{ \begin{array}{l} \frac{\partial K}{\partial \rho_{e}}={\sum_{e=1}^{N}p\cdot \left(\rho_{e}\right)}^{p-1}\cdot k_{e}\\ \frac{\partial M}{\partial \rho_{e}}={\sum_{e=1}^{N}p\cdot \left(\rho_{e}\right)}^{p-1}\cdot m_{e}\\ \frac{\partial \lambda_{m}}{\partial \rho_{e}}=\delta_{m}^{T}\cdot \left(\frac{\partial K}{\partial \rho_{e}}-\lambda_{m}\cdot \frac{\partial M}{\partial \rho_{e}}\right)\cdot \delta_{m} \end{array}\right.$$

Differentiating the single-objective function *C* with respect to design variables *ρ_e_*, we can obtain

(26) $$\begin{array}{l} \frac{\partial C}{\partial \rho_{e}}=\frac{\partial \left(\sum_{i=1}^{3}\sum_{j=1}^{3}{\tilde{U}}_{j}^{T}\cdot K\cdot U_{i}\right)}{\partial \rho_{e}}=\frac{\partial \sum_{j=1}^{3}{\tilde{U}}_{j}^{T}}{\partial \rho_{e}}\cdot K\cdot \sum_{i=1}^{3}U_{i}+\sum_{j=1}^{3}{\tilde{U}}_{j}^{T}\cdot \frac{\partial K}{\partial \rho_{e}}\cdot \sum_{i=1}^{3}U_{i}+\sum_{j=1}^{3}{\tilde{U}}_{j}^{T}\cdot K\cdot \frac{\partial \sum_{i=1}^{3}U_{i}}{\partial \rho_{e}}\\ =\left(\frac{\partial \sum_{j=1}^{3}{\tilde{U}}_{j}^{T}}{\partial \rho_{e}}\cdot K+\sum_{j=1}^{3}{\tilde{U}}_{j}^{T}\cdot \frac{\partial K}{\partial \rho_{e}}\right)\cdot \sum_{i=1}^{3}U_{i}+\sum_{j=1}^{3}{\tilde{U}}_{j}^{T}\cdot \left(\frac{\partial K}{\partial \rho_{e}}\cdot \sum_{i=1}^{3}U_{i}+K\cdot \frac{\partial \sum_{i=1}^{3}U_{i}}{\partial \rho_{e}}\right)-\sum_{j=1}^{3}{\tilde{U}}_{j}^{T}\cdot \frac{\partial K}{\partial \rho_{e}}\cdot \sum_{i=1}^{3}U_{i} \end{array}$$

Substituting $K=\sum_{e=1}^{N}\rho_{e}{}^{p}\cdot K_{e}$ and (25) into (26), the objective function *C* is given by

(27) $$\frac{\partial C}{\partial \rho_{e}}=\sum_{e=1}^{N}\sum_{i=1}^{3}\sum_{j=1}^{3}p\cdot {\tilde{U}}_{j}^{T}\cdot \rho_{e}{}^{p-1}\cdot K_{e}\cdot U_{i}$$

Note that the multi-objective function flexibility of integrated *F* can be described as

(28) $$\frac{\partial F}{\partial \rho }=-\omega \cdot \sum_{e=1}^{N}\sum_{i=1}^{3}\sum_{j=1}^{3}p\cdot {\tilde{U}}_{j}^{T}\cdot \rho_{e}{}^{p-1}\cdot K_{e}\cdot U_{i}+\frac{\left(1-\omega \right)\cdot \Lambda_{0}\cdot {\left(\Lambda -\lambda_{0}\right)}^{2}}{\Lambda^{2}\cdot s}\cdot \sum_{e=1}^{N}\sum_{m=1}^{7}\frac{\omega_{j}}{{\left(\lambda_{m}-\lambda_{0}\right)}^{2}}\cdot \frac{\partial \lambda_{m}}{\partial \rho_{e}}$$

So the optimization criteria can be derived by Equation (23) that consists of an objective function and two constraint conditions. The optimization criteria thus can be rewritten as

(29) $$\begin{array}{l} Find:Ρ={\left\{\rho_{1},\rho_{2},\rho_{3},\ldots ,\rho_{i}\right\}}^{T},i=1,2,\ldots ,m\\ \underset{\rho }{\min}F(\rho )={\left\{\omega^{2}\cdot {\left[\sum_{i=1}^{m}\omega_{i}\cdot \frac{C^{\prime }(\rho_{i})-C_{i}^{\min}}{C_{i}^{\max}-C_{i}^{\min}}\right]}^{2}+{\left(1-\omega \right)}^{2}\cdot {\left[\frac{\Lambda_{\max}-\Lambda^{\prime }(\rho_{i})}{\Lambda_{\max}-\Lambda_{\min}}\right]}^{2}\right\}}^{\frac{1}{2}},\\ s.t.\{\begin{array}{cc} V=f\cdot V_{0}=\sum_{i=1}^{m}\sum_{j=1}^{n}v_{ij}\cdot \rho_{i}\leq f_{0}\cdot V_{0}, & i=1,2,\ldots ,m,j=1,2,\ldots ,n\\ C^{\prime }(\rho_{i})=\frac{\sum_{i=1}^{m}\sum_{j=1}^{n}d_{ij}\cdot C(\rho_{i})}{\sum_{i=1}^{m}\sum_{j=1}^{n}d_{ij}}, & 0<\rho_{\min}\leq \rho_{i}\leq \rho_{\max}\leq 1. \end{array} \end{array}$$

where *m* is the number of all remained elements, *n* is the number of elements in sub-domains, *i* is the sub-domain number, *j* is the element number in sub-domains, *V* is the total volume of all remained elements, *v_ij_* is the volume of the *j*th element in the *i*th sub-domain, *f* is the volume fraction, *V*_0_ is the initial volume, *ρ*_min_ and *ρ*_max_ are the minimum and maximum value of the element-relative density respectively, *C*′ denotes the flexibility after filtering. *r_ij_* denotes the distance between the centers of the *i*th element and the *j*th element, *r*_min_ is a default radius of a circular subdomain Ω*_i_* centered at the center of a element, which determines the influence domain as the sensitivity varies*,* and *d_ij_* is the corresponding weight factor defined by *d_ij_* = *r*_min_ − *r_ij_*.

The iterative formula based on the multi-objective optimization criteria can be written as

(30) $$\rho_{i}^{k}=\{\begin{array}{cc} {\left[F_{i}^{k-1}(\rho )\right]}^{\tau }\cdot \rho_{i}^{k-1}, & \rho_{\min}<\rho_{i}^{k}<\rho_{\max}\\ \rho_{\min}, & \rho_{i}^{k}\leq \rho_{\min}\\ \rho_{\max}, & \rho_{i}^{k}\geq \rho_{\max}. \end{array}$$

where *k* is the iteration number, *τ* is the damping coefficient which guarantees the stability and convergence. The optimization process will converge, as the relative error *ξ* of two adjacent iterations is less than a convergence accuracy *ξ*_max_, which is defined as

(31) $$\xi =\frac{\left|\sum_{i=1}^{m}C_{k}-\sum_{i=1}^{m}C_{k-1}\right|}{\sum_{i=1}^{m}C_{k-1}}\leq \xi_{\max}$$

## 4. Multi-Objective Topology Optimization and Solution

### 4.1. Topology Optimization in Load Cases of a Multi-Objective Prototype Mechanism

Based on the 3-PRR planar fully CPMs, we sketch a holonomic triangular plate material to satisfy the isomorphic property with flexible hinge by slicing some regular region. Supposing that the elastic modulus of steel plate *E* is 21,000 Mpa, and Poisson’s ratio *μ* is 0.3, and material density *ρ* is 7900 kg/m^3^, and plate thickness is 8 mm, and volume fraction *f*_0_ is 0.4. Adopting 5510 triangle elements and 3029 nodes to fill the design region, the holonomic design system can be obtained.

**Figure 3:**
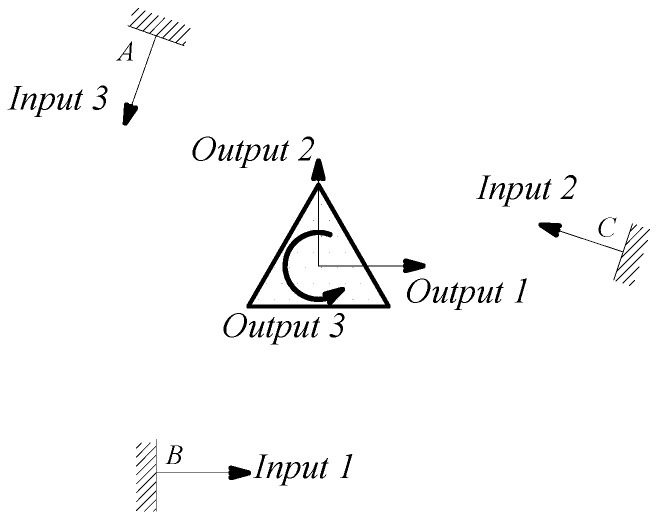
3-PRR planar fully CPMs topology optimization with load case.

**Figure 4:**
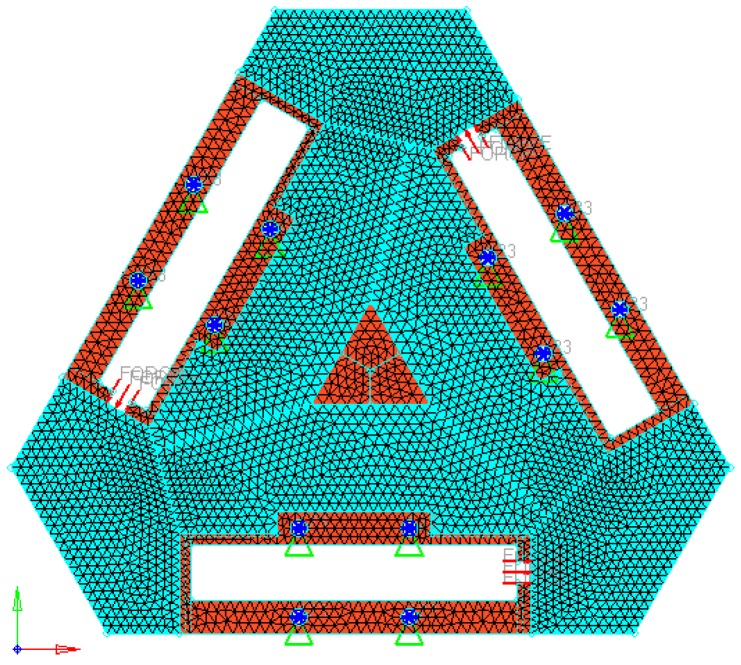
Continuum structure of preprocessing topology optimization model.

The linkage between moving platform and limbs has intrinsically been changed, shown in Figure 3. The post-optimality mechanism topology in load case is in free-hinged mode, satisfied with the 3-PRR fully CPMs. The finite element model was solved for each load case, and the von Mises stress distribution was obtained for each load case as the index for material removal (Xie and Steven [21]). The micro/nano-scale direct driver can be placed in the irregular design region whose scope is constrained to the driver’s build-in size. The prototypical model is built in 3-D software Solidworks^®^ (Dassault Systèmes SolidWorks Corp, Concord, MA, USA), and preprocessed in CAE software Hyperworks^®^ include fine removing, material element attributes assignment and input load case. The result is shown in Figure 4.

A detailed step-by-step algorithm of the multi-objective topology optimization method implemented for the CPM-type planar continuum structure is given as follows.

Step 1: Discretize the origin design domain with a densely finite element mesh as Figure 4.

Step 2: Define all boundary constraints and apply load conditions according to SIMP model as Equation (18).

Step 3: Assign an initial value to the design variables.

Step 4: Construct the relationship between flexibility C and frequency Λ as Equations (20), (22) and (23).

Step 5: Carry out a linear finite element analysis of the continuum structure.

Step 6: Differentiate the multi-objective formulation for all flexibility analysis as Equations (24), (27) and (28).

Step 7: Calculate the sensitivity of constraint function for all remaining elements as Equation (25).

Step 8: Calculate the optimization criteria as Equation (29) and update the design variables as Equation (30). Elements will be removed if they satisfy Equation (30).

Step 9: If the total volume V of all the remaining elements satisfies the volume constraint as Equation (29) and flexibility C satisfy Equation (31), the optimization process will terminate. Otherwise, the process will return to Step 4.

### 4.2. The Isomorphic Prototype of 3-PRR Planar Fully CPM

For the removed continuum structure in Hyperworks^®^, the attributes of selected material are set as material type, stainless steel 304#, Young’s modulus 2.05 × 10^−5^ F/mm^2^, Poisson’s ratio 0.3, material density 7.85 × 10^−9^ t/mm^3^, and the load 1 KN.

**Figure 5:**
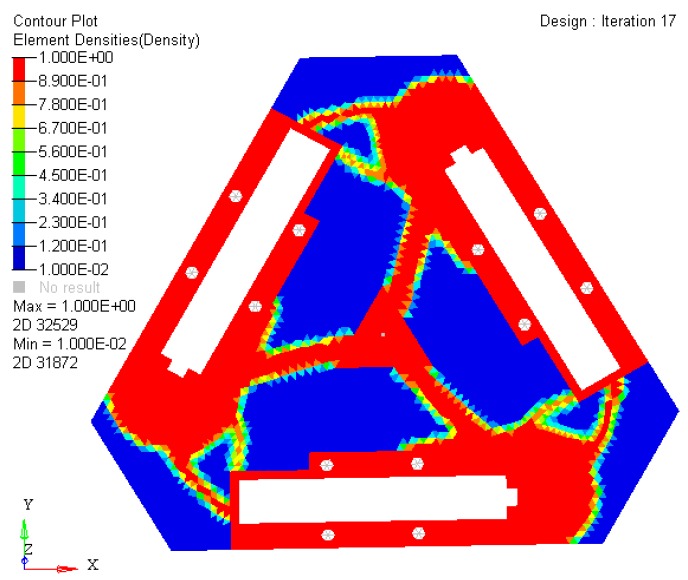
Topology optimizing in Hyperworks with prototype of 3-PRR planar fully CPMs.

**Figure 6:**
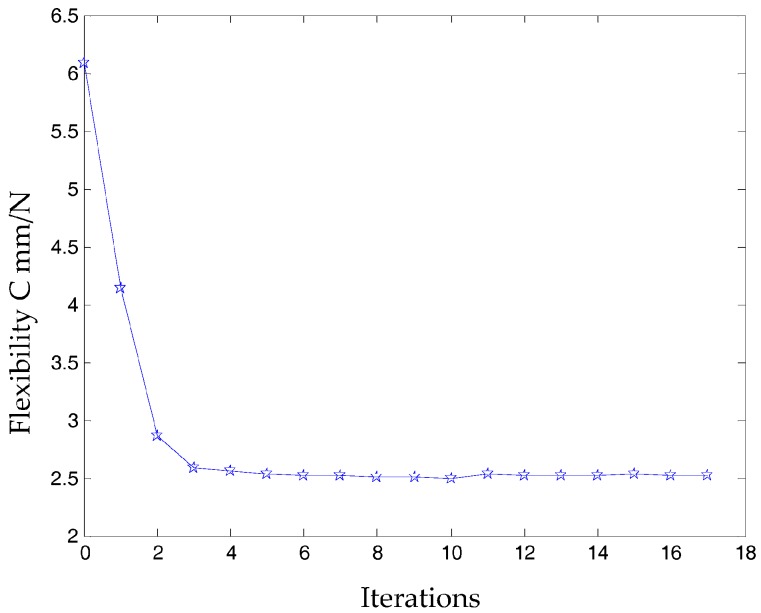
Topology optimization iteration procedure of 3-PRR planar fully CPM.

According to the continuous mapping topology optimization SIMP model defined as Equation (18), the material assignment in planar fully CPM prototype can be obtained by removing as shown in Figure 5. The reserved red region in the selected material field is filled with material with the density of 1, except for the non-design redundant material field. The blue region is filled with material with the density of zero, and should be removed from the redundant material field. The flexibility of objective function is shown in Figure 6, which converges fast after only 5 iterative steps.

### 4.3. The Analysis and Simulation Result on Topology Optimization

**Figure 7:**
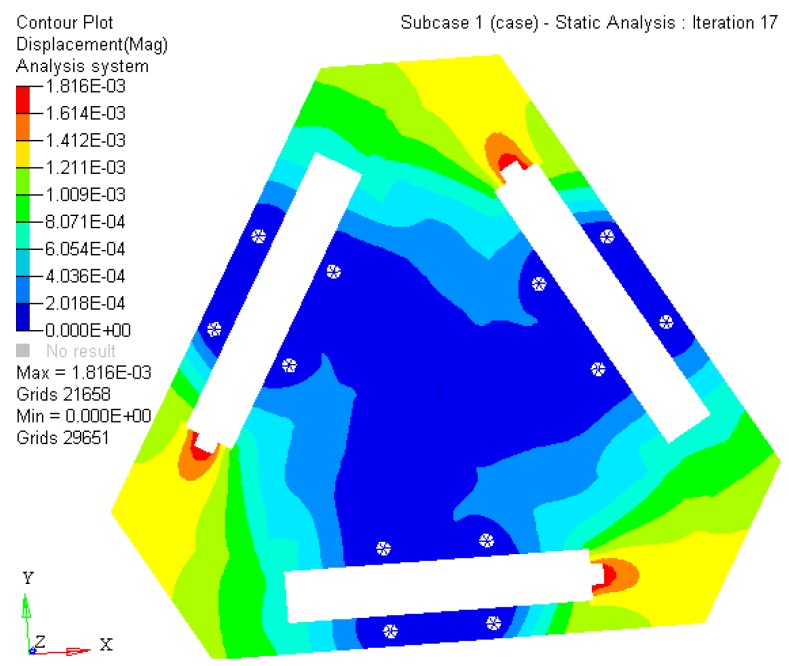
The stress distribution with the prototype of 3-PRR planar fully CPMs.

The CAD model from topology optimization of 3-PRR planar fully CPM is imported into Solidworks^®^, and also can be used in Hyperworks^®^ for kinematostatic simulation associated with processed results. The result of stress distribution is shown in Figure 7, and the maximum stress is centered in the outputs of drivers with the value of 29.64 MPa, less than the material yield strength.

Comparing the initial design mechanism parameters with optimized mechanism as shown in Table 1, stress distribution under different boundary constraints in the same load case can be obtained.

**Figure 8:**
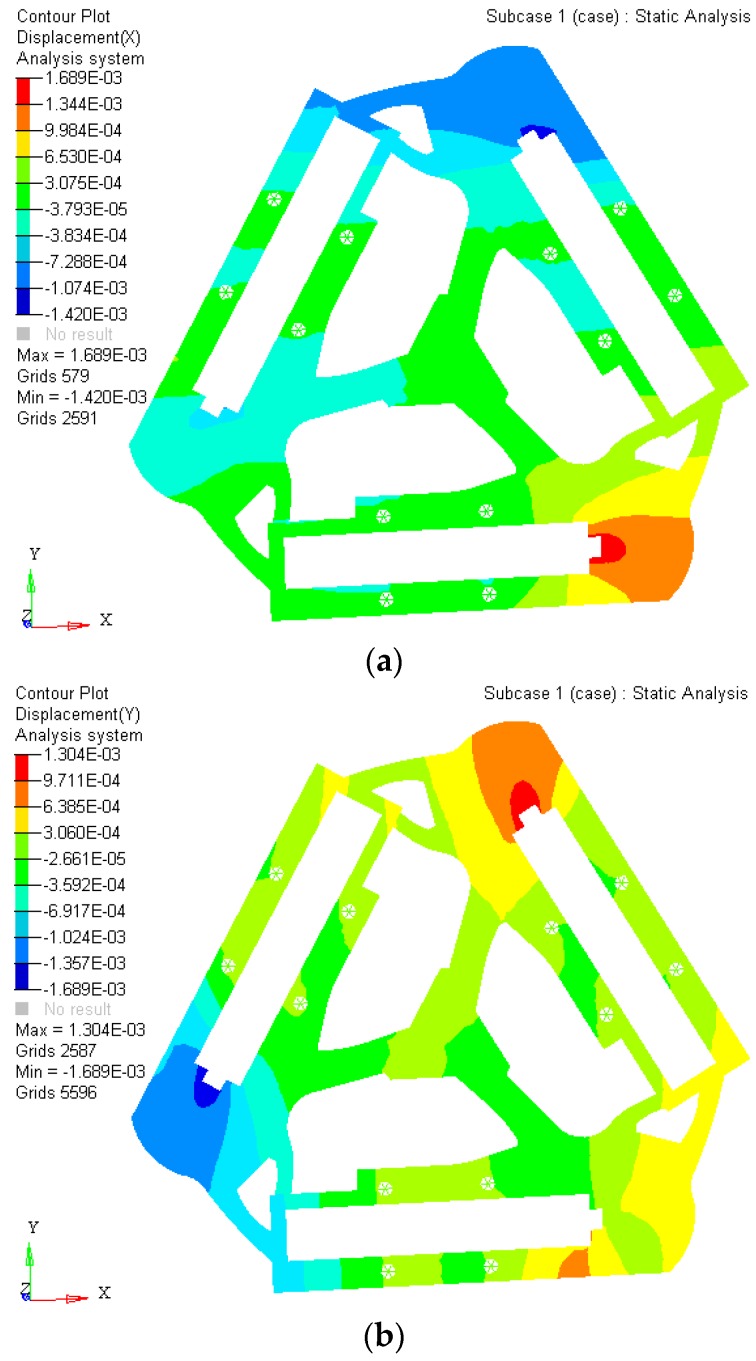

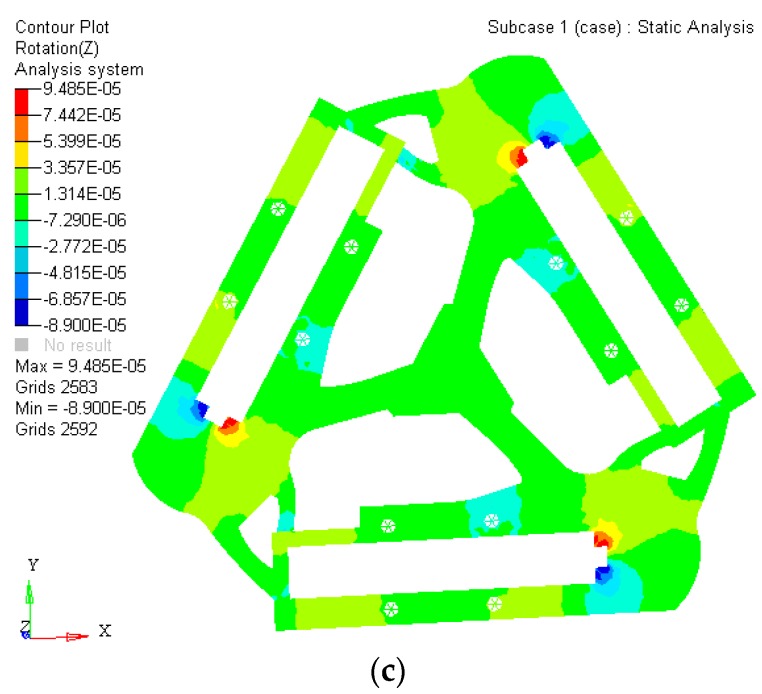
The kinematostatic characteristics of optimized material structure (**a**) the displacement of optimized material structure with direction *x*; (**b**) the displacement of optimized material structure with direction *y*; (**c**) the displacement of optimized material structure with rotational directional *z*.

The experiment results indicate that the maximum static stress of the optimized mechanism decreases by 47% of the initial unoptimized mechanism, and the minimum static stress of the post-optimality increases too. The material static stress homogeneity distribution is obviously better than the original mechanism. The kinematostatic characteristics of optimized material structure with the prototype of 3-PRR planar fully CPMs are shown in Figure 8, including the displacement of direction *x*-*y* and rotational directional *z* respectively.

**Figure 9:**
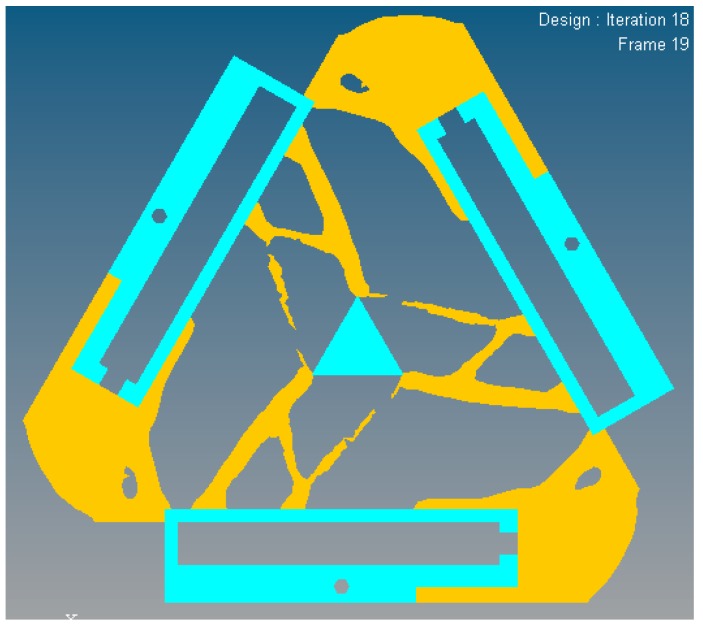
The material distribution of optimized topology mechanism in static load case.

**Figure 10:**
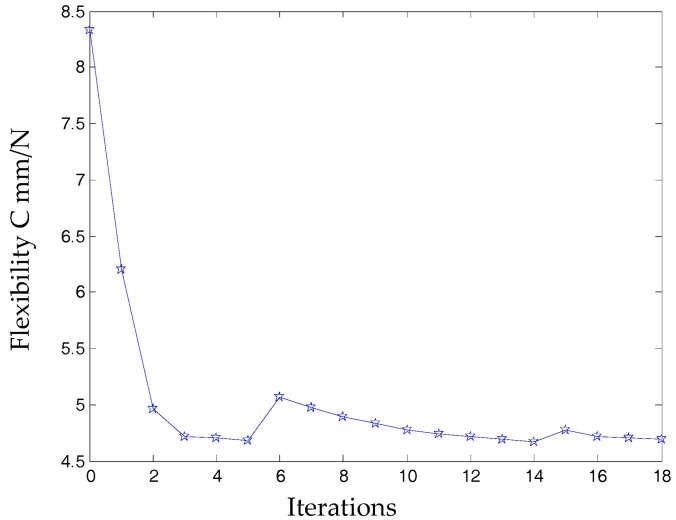
The objective function under static topological optimization conditions in iterations.

In this case, a static load of 1000 N is applied to each terminal of the optimized continuum structure with the prototype of 3-PRR planar fully CPM respectively. According to the mapping relation of input and output shown in Figure 3, the output displacements of direction *x*-*y* and rotational directional *z* are defined in the moving platform. After the topological optimization procedure with the flexibility minimizing as objective function and volume ratios as constraint conditions, the material element distribution and objective function iterations can be obtained as shown in Figure 9 and Figure 10, which converges after 18 iterations.

In Figure 9, the nattier blue pieces are a no-design region representing boundary constraint conditions and the piezoelectric ceramic actuator fixed mount. The yellow pieces are constituted of reserved elements with material density of 1 or close to 1. After optimized iterations, the maximum and minimum flexibility parameters of the optimized material structure with the prototype of 3-PRR planar fully CPMs are shown in Table 2.

**Figure 11:**
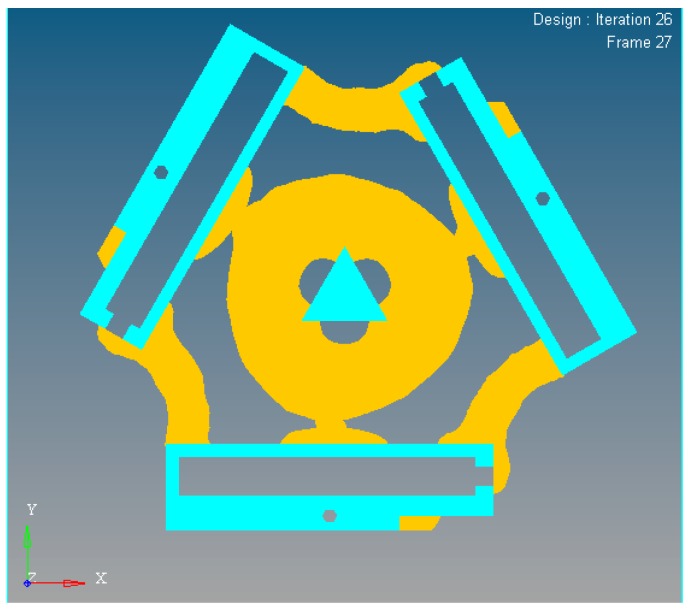
The material element distribution of dynamics topological optimization results.

**Figure 12:**
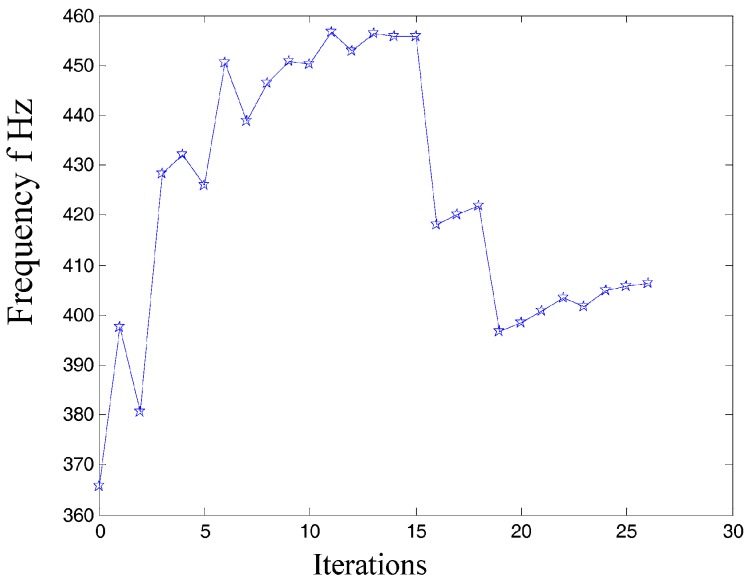
The objective function iteration of dynamics topological optimization results.

In the dynamics topological optimization iterations with first-order inherent frequency as objective function and volume ratios as constraint conditions shown as Figure 3, the material element distribution and objective function iterative figures after 26 iterations optimization can be obtained, as shown in Figure 11 and Figure 12 respectively.

The first 3 orders’ inherent frequency parameters of optimization material structure with 3-PRR fully CPMs are shown in Table 3.

**Figure 13:**
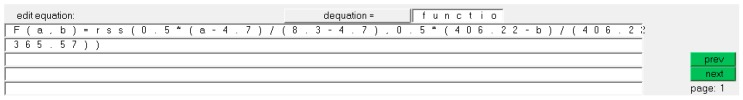
The user-defined integrated multi-objective function.

The inherent vibration characteristic of mechanism is modal, and it is especially important for micro/nano manufacturing. The modal analysis of optimized continuum structure mechanism is carried out under the same input load case and boundary constraint conditions with the software tool of Optistruct^®^. The integrated objective function is refined for the new optimized continuum structure mechanism with 3-PRR planar fully CPMs prototype. The first 6 modes of vibration modals are validated by self-weighted compromise programming with mean frequency method in user-defined function palette of Optistruct^®^. With the prototype of multi-objective integrated topological optimization Function (23), the integrated objective function is described as (32) with the maximum and minimum flexibility of static single objective topology optimization listed in Table 2 and maximum and minimum inherent frequency of the first 3 orders of dynamics single objective topology optimization listed in Table 3. The results of integrated objective functions are defined as (32)

(32) $$\underset{\rho }{\min}F(\rho )={\left\{{\left(\frac{1}{2}\right)}^{2}\cdot {\left[\sum_{i=1}^{m}\left(\frac{1}{2}\right)\cdot \frac{C_{i}\left(\rho \right)-4.7}{8.3-4.7}\right]}^{2}+{\left(\frac{1}{2}\right)}^{2}\cdot {\left[\frac{406.22-\Lambda \left(\rho \right)}{406.22-365.57}\right]}^{2}\right\}}^{\frac{1}{2}}$$

where *i* = 1 denotes single load case, we can define *ω_i_* = 1, and the multi-objective design variables in topology optimization as flexibility *C*_1_(*ρ*) and 1-order inherent frequency *Λ*(*ρ*), the maximum flexibility $C_{i}^{\max}$ before static optimization with value of 8.3 mm/N, the minimum flexibility $C_{i}^{\min}$ after static optimization with value of 4.7 mm/N, the maximum inherent frequency *Λ*_max_ after dynamics optimization with value of 406.22 Hz, the minimum inherent frequency *Λ*_min_ before dynamics optimization with value of 365.57 Hz. According to the *rss* function in Optistruct^®^, the integrated multi-objective optimization function is imported in the dequation palette as shown in Figure 13, substituting (a, b) to *C*_1_(*ρ*) and *Λ*_max_ respectively. So the minimum of integrated objective function *F*(a, b) and volume ratio constraint conditions are solved in module of Optistruct^®^, and the result is shown in Equation (33)

(33) $$F(a,b)=rss\left(0.5\cdot \left(\frac{a-4.7}{8.3-4.7}\right),0.5\cdot \left(\frac{406.22-b}{406.22-365.57}\right)\right)$$

**Figure 14:**
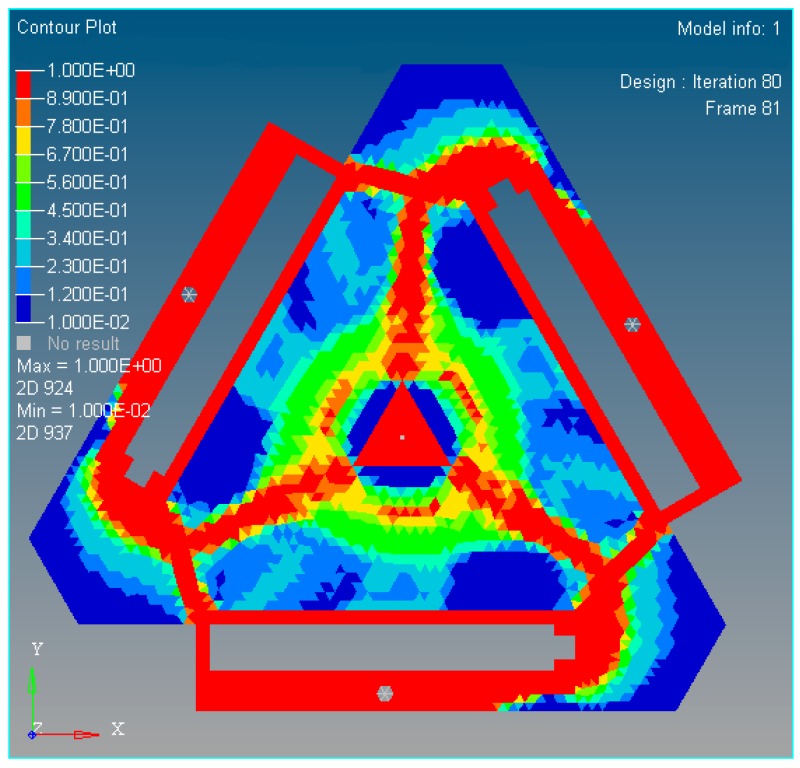
The multi-objective function topological optimization results of the new mechanism.

**Figure 15:**
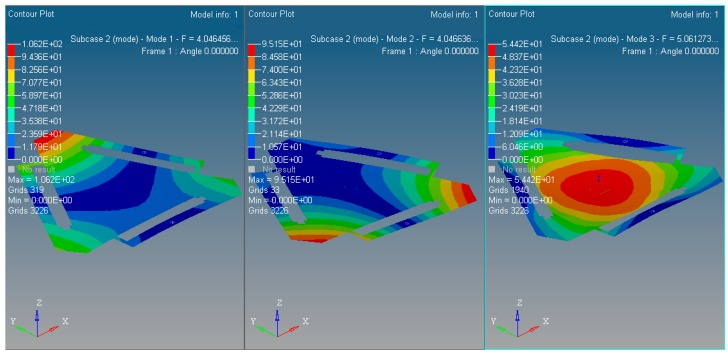
The 1st~3rd order modal shapes of the multi-objective optimized with the new mechanism.

**Figure 16:**
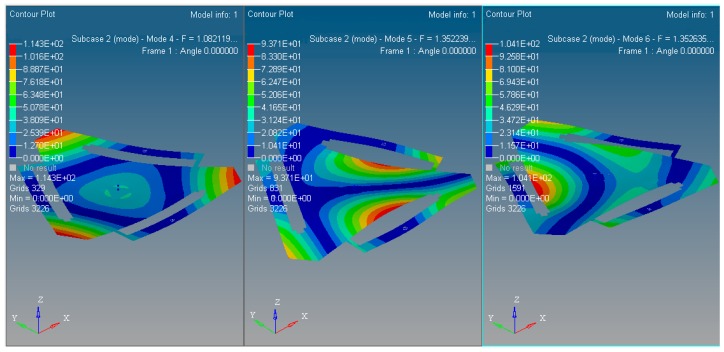
The 4th~6th order modal shapes of the multi-objective optimized with the new mechanism.

**Figure 17:**
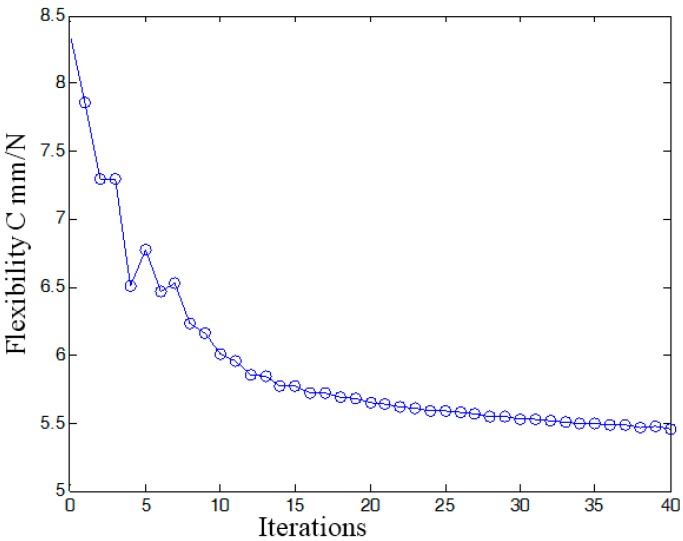
The flexibility iterative times during the multi-objective topology optimization.

**Figure 18:**
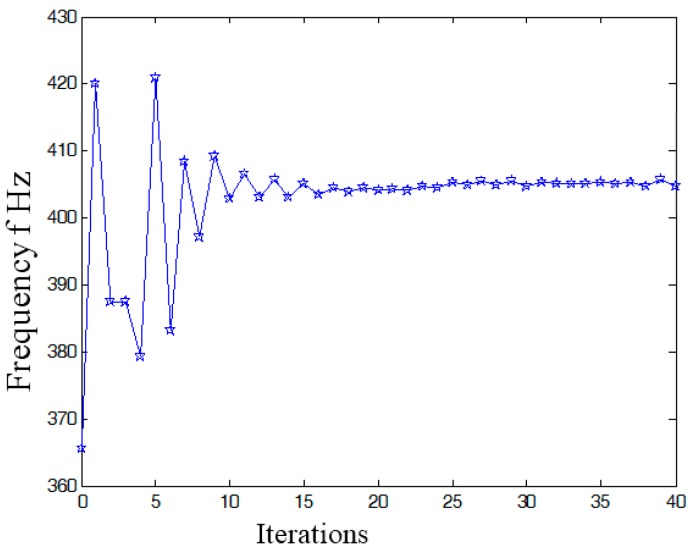
The first order inherent frequency iteration during the multi-objective topology optimization.

In summary, the optimized material structure model with prototype of 3-PRR planar fully CPMs carries out 40 optimizing iterations. The figures of multi-objective topology optimization results, the first 6 orders’ modal shapes, minimum flexibility iterations, and maximum frequency iterations can be obtained as shown in Figure 14, Figure 15, Figure 16, Figure 17 and Figure 18. Comparing Figure 18 of the first order inherent frequency iteration during the multi-objective topology optimization with that of Figure 12, the iterations are more stable and easily convergent by mean frequency method.

Figure 14 shows the optimization result of flexibility *C*, inherent frequency *Λ*, and volume fraction *f*. In Figure 17, the flexibility increases as the material is gradually removed from the design domain. When the total volume of the remaining elements reaches the objective volume, the flexibility goes to 5.489 mm/N and the inherent frequency stabilizes at around 405.5 Hz shown in Figure 18. It performs better than previous 3-PRR planar fully CPM limb-linked structure, which is attributed to the self-adjusting densities of all the elements in the optimization iterations.

The multi-objective optimization method obtains 40% of the initial volume (*f*_0_ = 0.4) at the 40th iteration, and the volume fraction *f* reaches 0.3836. When *f* is equal to 0.55, the optimization only needs 26 iterations. Modal shapes of all the remaining elements are displayed in sub-graphs of Figure 15 and Figure 16. The minimum and maximum flexibilities, the first 3 inherent frequencies and modal shape calculated after optimization are shown in Table 4.

The modal analysis results of the optimized material prototype as the 3-PRR planar fully CPMs show that low-order inherent frequency has been significantly improved more than the original status. This method can suppress the mechanism vibration and avoid losing precision positioning kinematics even resonance phenomenon. Meanwhile, the result of vibration-inherent frequency and relevant modal synthesis will provide a feasible basis for system synthesis (structural choice, dimension and topology optimization).

## 5. Experiments and Results

### 5.1. The Topology Optimization Kinematics Parameters Calculation

According to the differential Jacobian matrix in Equation (15), the kinematics analysis of optimized continuum structure mechanism with planar CPMs prototype can be initialized with the values shown in Table 5.

Substituting the initial values to (15), the Q matrix can be obtained as

(34) $$Q=\left(\begin{array}{ccc} -1.0000 & -1.7321 & 0\\ 3.7321 & 1.0000 & -33.4607\\ 0.5774 & -1.0000 & -19.3185 \end{array}\right)$$

**Figure 19:**
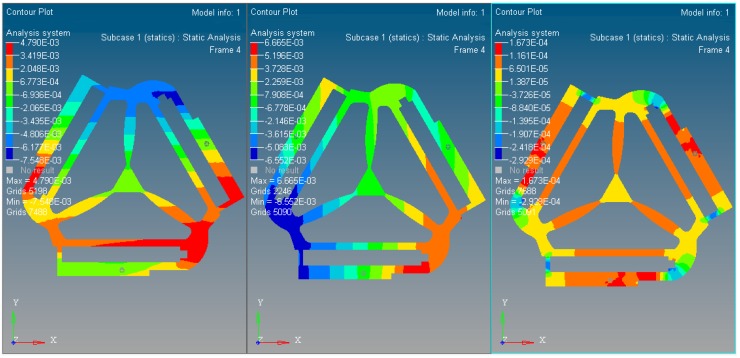
The kinematostatic characteristics simulation results by Hyperworks^®^.

Then the differential kinematics Jacobian J can be obtained as

(35) $$J=\left(\begin{array}{ccc} 1.3660 & 0.8660 & -1.5000\\ -1.3660 & -0.5000 & 0.8660\\ 0.1115 & 0.0518 & -0.1414 \end{array}\right)$$

where the displacements of the limb satisfies the conditions of [Δ*p*_1_ Δ*p*_2_ Δ*p*_3_]*^T^* = [5 × 10^−4^ 5 × 10^−4^ 5 × 10^−4^]*^T^* (unit: mm) with [Δ*x* Δ*y* Δ*δ*]*^T^* = *J*·[Δ*p*_1_ Δ*p*_2_ Δ*p*_3_]*^T^*. 3 DOF parameter can be obtained as [Δ*x* Δ*y* Δ*δ*]*^T^* = [3.7 × 10^−4^ −5 × 10^−4^ 1.1 × 10^−4^]*^T^*. Importing the topology optimized results to SolidWorks^®^ for further smooth processing, the kinematostatic characteristics are simulated in Hyperworks^®^ as shown in Figure 19.

### 5.2. The Micro/nano Actuator Construction

**Figure 20:**
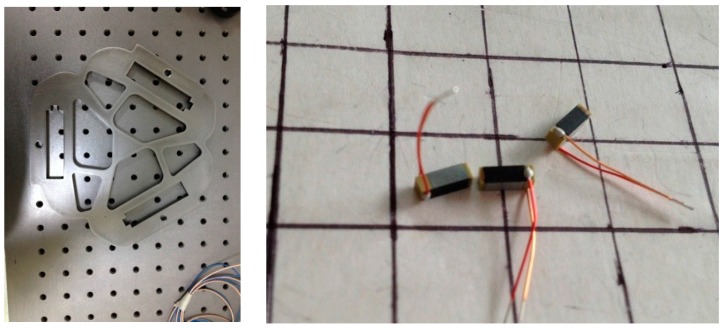
The physical model of the optimized material and PZT actuator.

With the results of optimized mechanism as 3-PRR planar CPMs prototype, we obtain the physical model by the configuration of wire-electrode cutting 45 stainless steel as shown in Figure 20. The piezoelectric ceramics actuator (PZT) with a type of piezoelectric bimorph is mounted in the fixed installation areas, which constructs a new non-hinge planar fully CPM of a holonomic material, and the parameters are listed in Table 6. The positioning control is implemented with three PZT actuator pairs applied to each mounting slot.

The holonomic architecture of the micro/nano actuator includes a PC-Based control system, driving power, PZT actuator and a displacement detection device. The control system is used with a high-precision numerical controller developed by us [35].

When a control voltage is applied to a PZT actuator, strain will generate on its surface due to the converse piezoelectric effect. The longitudinal bend and lateral torsion will be generated in the PZT actuators and can be converted to lumped planar torque exerted on the both sides of the actuators. The equivalent bending moment can be given as

(36) $$M_{piezo}^{active}(t)=\frac{b\cdot E_{p}\cdot d_{31}\cdot (t_{b}+t_{p})}{2}\cdot V_{a}(t)=M_{p}\cdot V_{a}(t)$$

where *M_p_* is the constant of the actuator, *b* is the width of PZT actuator, *t_b_* and *t_p_* are the slot and PZT thickness respectively, *V_a_*(*t*) is the control voltage.

**Figure 21:**
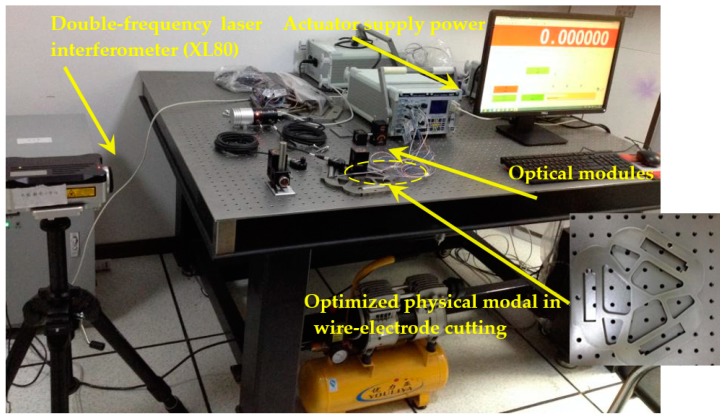
The control and measurement worktable for PZT-based micro/nano displacement.

The controller works in velocity or voltage mode, and the voltage output will be directly related to the velocity of actuator. Integrating the velocity to obtain the displacement will realize the micro/nano positioning. In order to avoid the possible interference during the experiment with ground vibration, air-flow and temperature fluctuation, the worktable and laser interferometer is placed in a laboratory of isolating external vibration disturbances, as shown in Figure 21.

**Figure 22:**
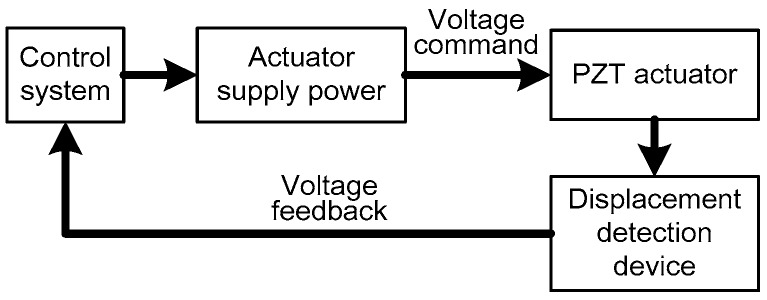
The control system to drive diagram in voltage mode.

**Figure 23:**
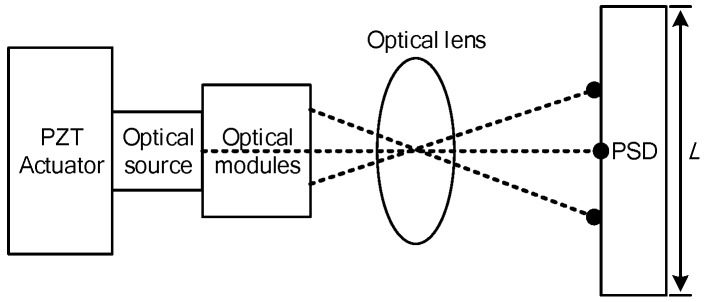
Configuration of fine positioning displacement detection device of PSD.

The control system transmits the 16-bit resolution analog voltage to PZT actuator, which realizes the continuum structure fine positioning displacement (*x* – *y* − *θ*) in the optimized moving platform as shown in Figure 22. The displacement detection device obtains the actual displacement data in voltage mode, and can feedback to the controller in need of positioning [36]. Compared with the commanded and actual position, the controller can adjust driving voltage deviation to achieve steady and fine positioning. Precise detection of PZT displacement is the basis of a PID closed-loop control [37]. Nevertheless, it is difficult to measure nano-level fine positioning displacement of PZT. Therefore, the PZT actuators are mounted in the wire-electrode cutting grooves in the optimized material with the 3-PRR prototype in this paper. The PZT actuators displacement is firstly measured in the form of optical signal by mounted optical modules, and then the optical signal is received by position sensitive detector (PSD) and converted to equivalent electrical signal feedbacking to the controller. The PZT fine-positioning steps should match the positioning resolution of the PSD, and amplify the fine displacement by optical lever, so the PZT displacement can be measured by PSD device. The micro/nano fine positioning displacement detection device is shown in Figure 23.

Let *x* be the distance of incident point of light and midpoint of PSD

(37) $$x=\frac{I_{2}-I_{1}}{I_{2}+I_{1}}\times L$$

where $I_{1}$ and $I_{2}$ are the two level output currents; *L* is the length of PSD, and *R* is selected to measure the voltages which can substitute the optical spot displacement in linear representation

(38) $$x=\frac{(I_{2}-I_{1})\cdot R}{(I_{2}+I_{1})\cdot R}\cdot L=\frac{V_{2}-V_{1}}{V_{2}-V_{1}}\cdot L$$

where $V_{1}$ and $V_{2}$ are $I_{1}$/$I_{2}$ and R to form output voltages of PSD, and actual displacement $x^{\prime }$ of PZT is

(39) $$x^{\prime }=\frac{V_{2}-V_{1}}{V_{2}+V_{1}}\cdot \frac{L}{\beta },$$

where β is the magnification of optical modules.

### 5.3. Experiment Validation

In this study, the PZT actuator power uses a DC-regulated power supply in an adjustable range with stepping value from 0.1 V to 1.0 V. Double frequency laser interferometer (type No. XL80, Renishaw Co. Ltd., Gloucestershire, UK) is used to measure the fine positioning in the displacement of direction *x*-*y* (XL80 linear optical modules) and rotational direction *z* (XL80 rotary shaft measuring angle optical modules). The linear measurement displacement resolution is 10^−6^ m, and the rotational resolution is 10^−6^ rad. The initial voltage is set to 20 V, and displacement step is *λ*/16 = 536 nm/16 = 33.5 nm with displacement detection device magnification 20. The micro displacement and rotation of PZT are measured many times by laser interferometer XL80 and the results are averaged to obtain the fine positioning displacement of direction *x*-*y* and rotational direction *z* respectively. Comparing the simulation with emulation experiment of PZT, the results are listed in Table 7.

It is found out that the experimental results are not compatible with the theoretical simulation results due to the manufacturing, actuator assembly errors, and so on. The difference between the results of this comparison can be understood that the integer part of the experimental results indicates submicron precision accuracy.

## 6. Conclusions

In this paper, a continuum structure isomorphic mechanism design method using multi-objective topology optimization is proposed, which is based on differential movement vector isomorphic mapping with a 3-PRR planar fully CPM prototype. Firstly, the Jacobian matrix of a 3-PRR planar fully CPM mapping relationship between input and output was obtained. Then, a homologous mechanism of 3-PRR in a holonomic material was constructed with vector isomorphic mapping. Upon this, multi-objective topology optimizing is applied to remove redundant material in the continuum structure by variable rejection rate. Thirdly, the simulation results are validated by the experiments on a planar fully CPM displacement system based on a PZT actuator. The experimental results of the optimized continuum structure mechanism are consistent with the kinematic characteristics of Equation (15) in the order of magnitude, which guarantees differential displacement accuracy and inherent vibration frequency. The structural feature and homology comparison of initial 3-PRR planar fully CPM and optimized continuum structure mechanisms are completely coincident in isomorphic properties. The experimental results show that fine positioning displacement actuated by the PZT of optimized planar fully CPMs are also consistent with theoretical expectations and simulation results, and the experiments simultaneously verify the repeatability in accuracy and configuration. In summary, we can draw the following conclusions.

(1) The SIMP optimization model is constructed with a mean frequency and compromise programming method. The multi-objective topology optimization weighted stiffness and frequency eigenvalue of 3-PRR planar fully CPMs prototype is solved by Optistruct^®^. The comparison analysis of static stiffness and first 3 orders’ inherent frequency before and after optimization show that the objects of maximizing static stiffness and vibration-inherent frequency is arrived. Using the mean frequency method, the 1-order frequency iterative diagram shows that amplitude of variation tends to be steady and convergent.
(2) The optimized 3-PRR planar fully CPMs prototype in continuum structure material is imported to Hyperworks^®^ for executing finite element static analysis. The displacement of 4.91 × 10^−4^ mm in *x* direction, −4.72 × 10^−4^ mm in *y* direction, and 2.39 × 10^−5^ rad in *z* angular is obtained by simulation, while the corresponding experimental displacement is 5.32 × 10^−4^ mm in *x* direction, −5.88 × 10^−4^ mm in *y* direction, and 3.91 × 10^−5^ rad in *z* angular. The sign and order of magnitude are consistent in all directions. It is validated that the integer part of the experimental results are sufficiently accurate to represent the design method of multi-objective topology optimization in a planar continuum structure.

## Figures and Tables

**Table 1:** Comparison of stress distribution before/after optimization.

Period	Maximum	Minimum
Before	55.95	8.851 × 10^−2^
After	29.64	1.289 × 10^−1^

Unit is MPa.

**Table 2:** The optimized material mechanism flexibility parameters in iterations.

Steps	Compliance: mm/N	Steps	Compliance: mm/N
0th	8.332893	9th	4.828077
1st	6.202367	12th	4.711885
2nd	4.960233	15th	4.778118
3rd	4.716350	18th	4.689774
6th	5.065805	-	-

**Table 3:** The optimized material mechanism flexibility parameters in iterations.

Steps	1-Order	2-Order	3-Order
0th	3.655654 × 10^2^	3.655925 × 10^2^	4.365313 × 10^2^
3rd	4.283030 × 10^2^	4.283868 × 10^2^	4.607935 × 10^2^
6th	4.504044 × 10^2^	4.504838 × 10^2^	4.568943 × 10^2^
9th	4.507617 × 10^2^	4.602106 × 10^2^	4.602923 × 10^2^
12th	4.529252 × 10^2^	4.529726 × 10^2^	4.641970 × 10^2^
15th	4.557257 × 10^2^	4.557837 × 10^2^	4.601292 × 10^2^
18th	4.218893 × 10^2^	4.220290 × 10^2^	4.321031 × 10^2^
21st	4.007571 × 10^2^	4.010012 × 10^2^	4.109230 × 10^2^
24th	4.048481 × 10^2^	4.051532 × 10^2^	4.104682 × 10^2^
26th	4.062230 × 10^2^	4.065501 × 10^2^	4.103355 × 10^2^

The first 3 orders inherent frequency (/Hz).

**Table 4:** The optimized mechanism flexibility, the first 3 inherent frequencies and the modal shapes recognition (unit: Frequency/Hz).

Steps	Flexibility (Compliance: mm/N)	1st Order	2nd Order	3rd Order
0th	8.332893	3.655654 × 10^2^	3.655925 × 10^2^	4.365313 × 10^2^
4th	6.514099	3.792490 × 10^2^	3.793375 × 10^2^	4.302357 × 10^2^
8th	6.228108	3.972276 × 10^2^	3.973315 × 10^2^	4.515919 × 10^2^
12th	5.853777	4.031293 × 10^2^	4.032470 × 10^2^	4.751472 × 10^2^
16th	5.722705	4.034842 × 10^2^	4.035777 × 10^2^	4.945665 × 10^2^
20th	5.652853	4.041327 × 10^2^	4.041886 × 10^2^	5.039851 × 10^2^
24th	5.591794	4.045151 × 10^2^	4.045541 × 10^2^	5.084239 × 10^2^
28th	5.551433	4.049462 × 10^2^	4.049620 × 10^2^	5.096651 × 10^2^
32nd	5.514682	4.050445 × 10^2^	4.050513 × 10^2^	5.090571 × 10^2^
36th	5.485535	4.051269 × 10^2^	4.051345 × 10^2^	5.072908 × 10^2^
40th	5.457566	4.046456 × 10^2^	4.046636 × 10^2^	5.061273 × 10^2^

The modal shape of 1st order substitutes longitudinal bending, and 2nd order substitute’s lateral torsion, and 3rd order substitutes longitudinal bending.

**Table 5:** The initial values of differential Jacobian matrix.

Parameters	$\varphi_{1}$	$\varphi_{2}$	$\varphi_{3}$	δ	e
Initial values (/rad, /mm)	$\frac{2}{3}\pi$	$\frac{13}{12}\pi$	$\frac{2}{3}\pi$	$\frac{1}{12}\pi$	8

**Table 6:** The parameters of PZT actuator.

Terms	Values
Type of actuator	RS47-5.9-0.8-1
Overall dimension *L* × *W* × *T* (mm)	47.2 × 5.9 × 0.8
Supply Voltage (DCV)	0–200
Bidirectional displacement (mm)	±1.8
Unidirectional output force (mN)	300
Maximum amplitude response	≥200
Electrostatic capacitance (nF)	44.8
Young’s modulus (*E_p_*)	3.8 × 10^9^ N/m^2^
Piezoelectric constant (*d*_31_)	−189 × 10^−^^12^ C/N

**Table 7:** Comparison results between the simulation and emulation with multi-objective optimized kinematics mechanism.

Direction	Simulation	Experiment
Displacement in *x*’	4.91 × 10^−4^ mm	5.32 × 10^−4^ mm
Displacement in *y*’	−4.72 × 10^−4^ mm	−5.88 × 10^−4^ mm
Rotation in *θ*_z_	2.39 × 10^−5^ rad	3.91 × 10^−5^ rad
